# Structural connectome gradients and their relationship to IQ in childhood

**DOI:** 10.3389/fnhum.2025.1688296

**Published:** 2025-11-24

**Authors:** Yoonmi Hong, Emil Cornea, Jessica B. Girault, Rebecca L. Stephens, Maria Bagonis, Mark Foster, Sun Hyung Kim, Juan Carlos Prieto, Martin A. Styner, John H. Gilmore

**Affiliations:** 1 Department of Psychiatry, University of North Carolina at Chapel Hill, Chapel Hill, NC, United States; 2 Carolina Institute for Developmental Disabilities, University of North Carolina at Chapel Hill, Chapel Hill, NC, United States; 3 Division of Neuroscience, National Institute of Neurological Disorders and Stroke (NINDS) at the NIH, Bethesda, MD, United States; 4 Department of Computer Science, University of North Carolina at Chapel Hill, Chapel Hill, NC, United States; 5 Department of Biomedical Engineering, University of Basel, Basel, Switzerland

**Keywords:** structural connectome, diffusion MRI, connectome gradient, graph convolutional neural, IQ prediction

## Abstract

The concept of connectome gradients, which represents the continuous spatial variation of brain connectivity, offers a robust framework for exploring the hierarchical organization of the cortex and its relationship with cognitive function. We hypothesize that structural gradients in frontal and parietal regions play a significant role in shaping individual cognitive abilities during early childhood. To evaluate this hypothesis, we identified macroscale structural connectome gradients in children aged 1–6 years, where the principal gradient exhibited a left-to-right axis, and the secondary gradient exhibited an anterior-to-posterior axis. Next, we employed machine learning approaches to predict the future cognitive outcomes assessed at ages 4, 6, and 8, specifically intelligence quotient (IQ), based on the structural connectome gradients measured at age 1. We achieved consistent and robust prediction results (mean Spearman's correlation > 0.25). The regional relevance maps highlighted regions in control network, and associated sensory processing networks. Our findings indicate that the structural connectome, which undergoes maturation during early childhood, plays a crucial role in the individual variability of IQ observed in early and middle childhood. Our approach underscores the utility of structural gradients as compact and interpretable representations of the brain's complex network architecture, effectively capturing individual differences that contribute to cognitive development.

## 1 Introduction

Early childhood is widely recognized as a critical period for the development of lifelong cognitive abilities and behaviors. Moreover, it is a crucial window for understanding and mitigating risk factors for neuropsychiatric disorders (Gilmore et al., 2018). Cognitive performance plays a key role in the academic and social adjustment of school children (Durlak et al., 2011; DeRosier and Lloyd, 2010; Racz et al., 2017). Identifying early imaging biomarkers of brain development can potentially predict and track cognitive trajectories, allowing for timely interventions to optimize learning outcomes and support. Studies have found significant associations between IQ and cortical attributes or microstructures. For example, there is evidence of positive correlations between IQ and cortical thickness (Narr et al., 2007; Karama et al., 2011), surface area (Girault et al., 2020), and mean occipital fractional anisotropy (FA) (Dubner et al., 2019). However, the specific pathways linking early brain network organization to subsequent cognitive outcomes remain largely unknown.

A continuous spatial representation of connectivity across the cortical surface, referred to as connectome gradients, provides a valuable framework for exploring the topographical organization of the cortex and its relationship to cognitive functions. A series of low-dimensional manifold representations can be obtained by non-linear dimensionality reduction techniques, so-called diffusion map embedding (Coifman et al., 2005; Margulies et al., 2016). These gradients provide interpretable, topographic summaries that capture distributed patterns of brain organization that may be missed by traditional node-based or graph centrality metrics.

An increasing body of research has shown that the functional connectome gradient runs from primary sensorimotor and visual to higher-order transmodal regions (Margulies et al., 2016; Xia et al., 2022; Paquola et al., 2019). Xia et al. (2023) demonstrated that the functional connectome gradient present at birth significantly predicts cognitive outcomes at the age two, highlighting its early role in neurodevelopment. Yang et al. (2024) showed that alterations in the brain's primary-to-transmodal functional connectome gradient are linked to the severity of white matter (WM) hyperintensities and partially account for the consequent decline in executive cognitive function. Further research has found that disruptions in functional connectome gradients across various conditions are associated with a range of cognitive deficits, suggesting that gradient dysfunction may serve as a biomarker of disease-related cognitive impairment (Li et al., 2025). Collectively, these studies establish functional connectome gradients as essential organizational frameworks of brain networks, with their integrity and development closely associated with diverse cognitive abilities.

In contrast to extensive investigations into functional connectome gradients, the understanding of structural connectome gradients and their relationship to cognition remains more limited. A substantial body of research has shown that the WM connectome is established very early in childhood and remains relatively stable thereafter (Gilmore et al., 2018; Bagonis et al., 2022; Hong et al., 2023). Diffusion magnetic resonance imaging (dMRI) is a non-invasive imaging technique that characterizes tissue microstructure and white matter tracts (Johansen-Berg and Behrens, 2013), allowing the computation of anatomical brain networks or structural connectomes (SC). Because the SC provides the anatomical constraints on functional connectivity and reflects the underlying white matter architecture established in early development, structural connectome gradients could offer a distinct and complementary perspective on cortical organization not captured by functional gradients alone. Notably, Park et al. reported that structural connectome gradients mature progressively during adolescence in ways that predict individual differences in cognitive functions, including intelligence (Park et al., 2021). However, it is still unknown whether structural connectome patterns in infancy can predict IQ at later ages, and specifically whether higher-order association networks implicated in adult intelligence are already the most predictive features during infancy.

Extensive research has established that higher-order association networks, particularly the frontoparietal network, cingulo-opercular network, and default-mode network, are consistently associated with cognitive ability and intelligence (Jung and Haier, 2007; Chang et al., 2019; Davis and Cabeza, 2015; Sheffield et al., 2015; Smallwood et al., 2021; Menon and Uddin, 2010). Girault et al. demonstrated that WM connectomes at birth can be used to predict the individual differences in 2-year cognitive performance using machine learning approaches, highlighting the importance of the WM connectome as an imaging biomarker of subsequent cognitive development (Girault et al., 2019). Previous studies based on graph centrality metrics have not found significant associations between the structural or functional connectome and cognitive abilities in early childhood (Bagonis et al., 2022; Jiang et al., 2023). This discrepancy suggests that traditional regional or graph centrality metrics may miss the distributed, low-rank topographic patterns that the structural gradients can capture. Although it is widely known that the SC is related to cognition and behavior in adults and adolescents (Seguin et al., 2020; Dhamala et al., 2021), little is known about their relationships in early childhood. Understanding when these network patterns emerge and whether they can predict later cognitive outcomes would have important implications for early identification and intervention (Ji et al., 2024).

We hypothesize that structural connectome gradients are detectable at age 1, show stability across early childhood, and predict later cognitive performance. Specifically, we propose that structural connectome gradients in regions important for cognitive function and intelligence, particularly the dorsolateral prefrontal cortex and superior parietal cortex (Jung and Haier, 2007; Deary et al., 2010; Basten et al., 2015), will be associated with individual cognitive differences in young children. Furthermore, we hypothesize that individual differences in the spatial organization of structural connectome gradients may correlate with variations in individual cognitive outcomes. These structural connectome patterns likely emerge within the first year of life, suggesting that interventions aimed at normalizing developmental trajectories may need to be implemented during very early childhood (Gilmore et al., 2020). To test these hypotheses, we aim to predict the individual's cognitive outcomes measured via the Stanford–Binet Intelligence scales from their structural gradients at age 1, using a graph convolutional neural network model where the input features are the structural gradients and the adjacency matrix is the whole brain SC. This approach underscores the potential of structural gradients as early predictors of cognitive development.

## 2 Materials and methods

### 2.1 Datasets

Multi-modal neuroimaging data from the University of North Carolina (UNC) early brain development study (EBDS) were used for this study. In this study, we included subjects who underwent brain scans at ages 1, 2, 4, and/or 6 years, along with cognitive assessments conducted at ages 4, 6, and 8 years. One twin from each twin pair was included in the study; twin A was selected when both twins had usable scans. Subjects were excluded if they had an abnormality on MRI or a major medical or surgical illness, including head injury or seizure disorder (see Supplementary Table 1). Here, the number of excluded diffusion weighted images (DWIs) and the number of DWIs with large translation (>1 mm) were employed as estimates of head motion and image quality. We additionally excluded the subjects if the number of excluded DWIs are greater than 14.

Structural T1w/T2w images and diffusion-weighted images (DWIs) were acquired using either a Siemens Allegra scanner or a Siemens Tim Trio scanner. T1-weighted images on the Siemens Allegra scanner were acquired using a 3-dimensional magnetization-prepared rapid acquisition gradient-echo (MPRAGE) sequence: TR = 1,880–1,900 ms, TE = 4.38 ms, flip angle = 7°, voxel size = 1 × 1 × 1 mm^3^. T2w images on the Allegra scanner were collected using a dual echo sequence: TR = 7,380–8,010 ms, TE1 = 20 ms, TE2 = 119 ms, flip angle = 150°, voxel size = 1.25 × 1.25 × 1.5 mm^3^. T1w images on the Tim Trio scanner were acquired using a lower echo time: TR = 1,900–1,940 ms, TE = 3.74 ms, flip angle = 7°, voxel size = 1 × 1 × 1 mm^3^. T2w images on the Tim Trio scanner were collected using a 3DT2 SPACE protocol: TR = 3,200 ms, TE = 406–497 ms, flip angle = 120°, voxel size = 1 × 1 × 1 mm^3^.

DWIs were obtained using both scanners following an identical protocol. This protocol utilized 42 unique gradient-sensitizing directions, uniformly distributed across the sphere, with a *b*-value of 1,000 s/mm^2^ in addition to seven *b* = 0 images. The following acquisition parameters were used: twice refocused spin echo, TR = 7680 ms, TE = 82 ms, flip angle = 90°, voxel size = 2 × 2 × 2 mm^3^.

**Figure 1:**
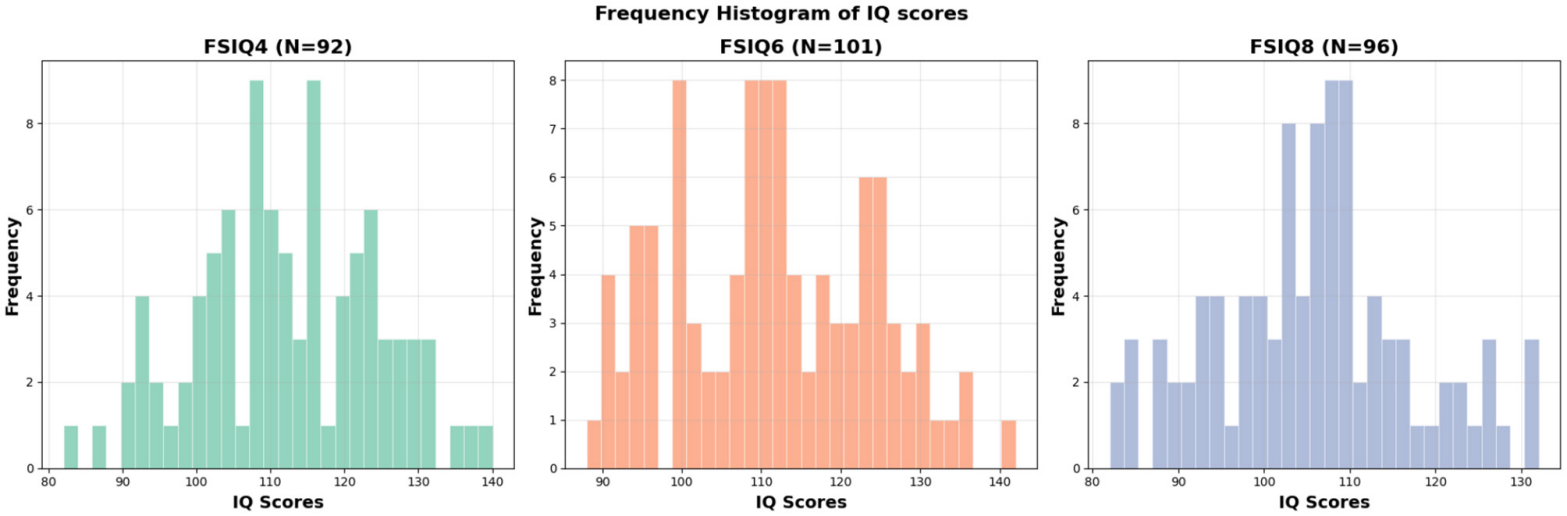
Histogram of FSIQs at different ages. FSIQ4: mean = 112.04, std = 12.34, FSIQ6: mean = 111.43, std = 12.33, FSIQ8: mean = 105.55, std = 11.54.

The cognitive performance of each subject was assessed by the 5th Edition of the Stanford–Binet Intelligence scales (Roid, 2003). The Stanford–Binet is a standardized set of assessments used to assess intelligence (IQ) across the lifespan. This study focuses primarily on the full-scale IQ (FSIQ) score, with exploratory analyses examining effects on abbreviated IQ (ABIQ), verbal IQ (VIQ), and nonverbal IQ (NVIQ). The composite scores generated from the Stanford–Binet have strong inter-rater reliability (ranging from 0.74 to 0.97 with a median of 0.90) and test–test reliability (correlations in the 0.80s and 0.90s). All of the scores utilized in this study are normalized standard scores, with means of 100 and standard deviations of 15. The distribution of FSIQ scores across ages 4, 6, and 8 years is presented in Figure 1. Detailed demographic and scan information for participants in the FSIQ prediction analysis can be found in Table 1.

**Table 1:** Participant demographic and scan information for subjects used for the IQ prediction.

**Variable**	**FSIQ4**	**FSIQ6**	**FSIQ8**
	**N=92**	**N=101**	**N=96**
	**Mean (SD)**	**Mean (SD)**	**Mean (SD)**
Age at scan (months)	12.95 (0.94)	12.95 (0.89)	12.93 (0.85)
Number of excluded DWIs	1.46 (1.84)	1.63 (2.05)	1.61 (1.94)
Number of DWIs with large translation (>1mm)	0.32 (0.77)	0.53 (1.42)	0.55 (1.42)
	*N*(%)	*N*(%)	*N*(%)
Allegra	62 (67%)	69 (68%)	65 (68%)
Tim Trio	30 (33%)	32 (32%)	31 (32%)
Sex, male	50 (54%)	54 (53%)	51 (53%)

### 2.2 Structural connectome processing

Diffusion MRI data were pre-processed using DTIPrep (Oguz et al., 2014) to correct eddy current and motion artifacts. DTIPrep also removed DWI volumes with significant motion artifacts. Both the T1w image and the white matter (WM) surface were brought into DWI space by applying rigid and non-linear transforms. Probabilistic tractography was initiated from each labeled vertex on the WM surface using probtrackx2 (Behrens et al., 2007). For each seed, 1,000 streamlines were generated, with a step size of 0.75 mm and a seed sphere sampling radius of 0.5 mm. More details can be found in our previous work (Hong et al., 2023). Connectome matrices with dimensions 148 × 148 from the Destrieux parcellation and 78 × 78 from the AAL parcellation were generated by counting the number of streamlines connecting each pair of regions. We note that a surface-based labeling with the AAL parcellation was created from voxel-level parcellation (Kim et al., 2005). The raw streamline counts were symmetrized. We then normalize the matrices so that the summation of the lower triangular parts equals to 1.

### 2.3 Connectome gradients

We computed SC gradients using BrainSpace toolbox (Vos de Wael et al., 2020). The SC matrix was converted to an affinity matrix using a cosine similarity, where the cosine similarity of two row vectors in SC is defined as


$$\cos(v_{i},v_{j})=\frac{v_{i}\cdot v_{j}}{\left\|v_{i}\|\|v_{j}\right\|}.$$ (1)

**Figure 2:**
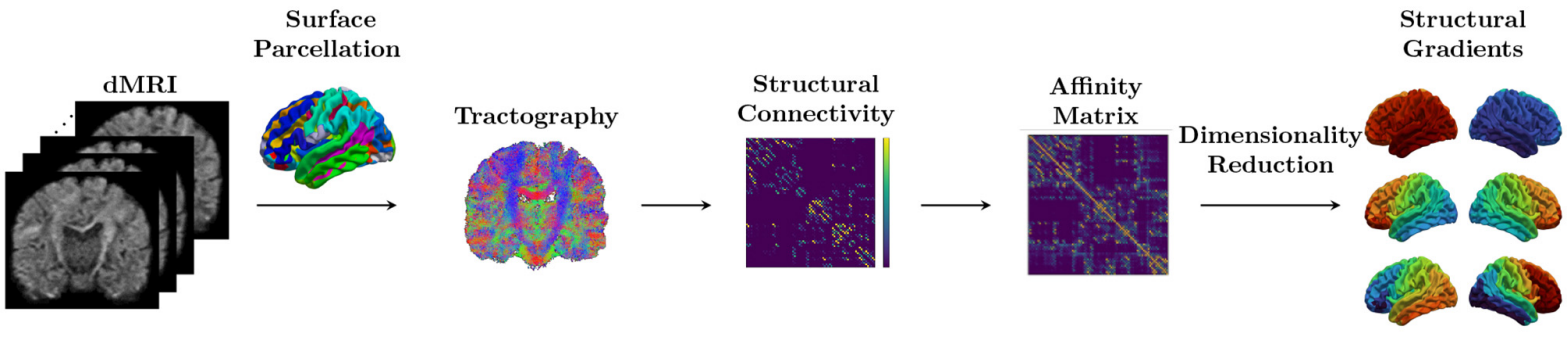
Schematic overview. The structural connectome (SC) matrix is generated from dMRI by counting the number of streamlines connecting each pair regions in surface parcellation. Diffusion map embedding, a non-linear dimensionality reduction technique, is applied to the affinity matrix computed from the SC. A set of gradient vectors was calculated and sorted based on their corresponding eigenvalues.

Then, the diffusion map embedding, a non-linear dimensionality reduction technique, was applied (Coifman et al., 2005). A set of gradient vectors was calculated and sorted based on their corresponding eigenvalues. The eigenvector corresponding to the largest eigenvalue is defined as the principal gradient. Since each subject's gradients represent relative distances and can be mapped to arbitrary axes, we applied Procrustes rotation approaches to align each subject's gradient vectors to the template (Langs et al., 2015). We created a reference template from an independent set of 10 subjects from same dataset at age 1 not included in our analysis. To correct the scanner batch effect, the computed SC gradients were harmonized using ComBat (Fortin et al., 2018) with gestational age at birth, sex, the number of DWIs with large motion translation, and the number of excluded DWIs as covariates. A schematic overview of the SC gradients generation is illustrated in Figure 2.

### 2.4 IQ prediction model

We applied a graph-convolutional neural network model (GCN) to predict each individual's IQ score from their SC gradients at age 1. The principal structural gradient vector as well as the secondary gradient were used as input features, and two layers of GCN with SC matrix as an adjacency matrix were applied to extract the latent vector. The final regression score was predicted with a multi-layer perceptron. A new loss function was employed to ensure that the subjects having similar cognitive scores had higher agreement in their prediction.

Our GCN model is based on a spectral graph convolutional operation. The input features are the structural gradients, and the adjacency matrix is the SC matrix. Let *A* be the weighted graph adjacency matrix and *N* be the number of nodes in the graph. Then graph Laplacian operator *L* can be defined as $L=I_{N\times N}-D^{-1/2}AD^{-1/2}$ with *I*_*N*×*N*_ being the identity matrix and *D* being the diagonal degree matrix $D_{ii}=\underset{j}{\sum }A_{ij}$. Let **H**^(*l*)^∈ℝ^*N*×*d*^^(*l*)^ be *l*-th layer features, where *d*^(*l*)^ is the number of features at *l*-th layer. The output features in GCN layers are updated as


$$H^{\left(l+1\right)}=\xi (\sum_{k=0}^{K}T_{k}(\tilde{L})H^{\left(l\right)}W_{k}^{\left(l\right)}),$$ (2)

where ξ is a non-linear activation function and ${\text{W}}_{k}^{\left(l\right)}\in {\mathbb{R}}^{d^{\left(l\right)}\times d^{\left(l+1\right)}}$ is the matrix of learnable parameters at *l*-th layer, representing *k*-th order Chebyshev polynomial coefficients. Here, $T_{k}\left(\tilde{L}\right)$ is the *k*-th order Chebyshev polynomial evaluated on the scaled graph Laplacian $\tilde{L}:=2L/\lambda_{\max}-I$ with λ_max_ being the maximal eigenvalue of *L*. Note that the spectral filters learned as *K*-th order Chebyshev polynomial coefficients are exactly *K*-localized and can be computed recursively (Defferrard et al., 2016).

Let **x**_*i*_ be an input SC gradient, **y**_*i*_ ground-truth IQ, and *f*(**x**_*i*_) predicted IQ. The loss function is the weighted sum of the mean-squared error and the Siamese loss which are defined as:


$$\begin{array}{l} L=L_{\text{MSE}}+w_{\text{siamese}}L_{\text{siamese}}, \end{array}$$ (3)

where


$$\begin{array}{lll} L_{\text{MSE}} & = & \underset{i}{\sum }|{\text{y}}_{i}-f\left({\text{x}}_{i}\right){|}^{2}, \end{array}$$ (4)

$$L_{\text{siamese}}=\sum_{i\neq j}[(y_{i}-y_{j})-(f(x_{i})-f(x_{j})){]}^{2}.$$ (5)

Here, we added a paired inter-subject difference loss $L_{\text{siamese}}$ to ensure that the difference of the predicted IQs across different subjects are comparable to the difference of ground-truth IQs. This regularization function helps to retain inter-subject heterogeneity, avoiding the case that the prediction converges to the group-averaged IQs.

We trained the prediction model with principal and secondary structural gradients and performed 10-fold cross-validation to evaluate the model and repeated the prediction 10 times with different training and test splits to mitigate the bias with respect to subject selection. To avoid information leakage, within each cross-validation fold, structural gradients from both training and test sets were aligned to the external reference generated in Subsection 2.3. Prediction accuracy was evaluated by mean absolute error (MAE), the Spearman's rank correlation, and the Pearson's correlation between the ground-truth score and the predicted score.

Notably, we applied ComBat harmonization to the combined training and testing data within each cross-validation fold. While best practice recommends applying ComBat separately to training and testing sets to prevent data leakage, our limited sample size (~10 per test fold) precluded reliable scanner effect estimation for individual test sets. This limitation is discussed further in Section 4.

### 2.5 Implementation details

We implemented our machine learning prediction model in PyTorch 1.7.0, and the model training and testing were performed on an NVIDIA Titan Xp GPU machine with CUDA 12.0. The initial learning rate was set to 0.005 and the ADAM optimizer was used. The weight for the Siamese loss was set to *w*_siamese_ = 10. The total number of training epochs was 300, with the model evaluated at the final epoch, rather than using early stopping or validation-based checkpointing. This choice was made to maximize the use of training data within each cross-validation fold given the limited sample size. The batch size was set to 10. The order of the Chebyshev polynomial in the GCN was set to *K* = 3. The training time was about 2 min, depending on the sample sizes (82–91 subjects) for each cross-validation fold.

We applied *z*-score normalization to each gradient component separately for each hemisphere, and performed min-max normalization on IQ scores, setting the minimum to 50 and the maximum to 150.

## 3 Results

### 3.1 Macroscale structural connectome gradients

**Figure 3:**
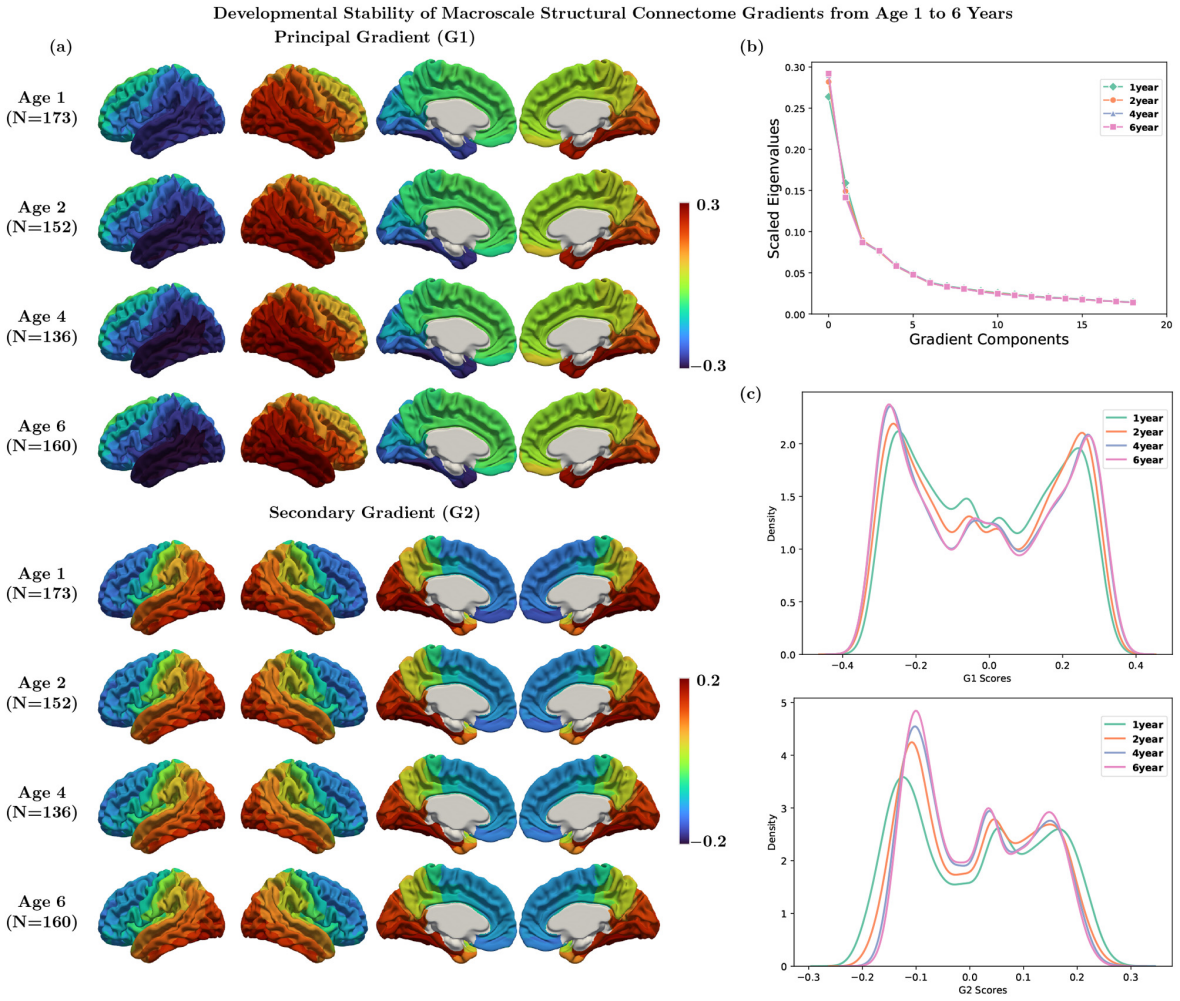
**(a)** Average structural gradients at each age using Destrieux parcellation. **(b)** Explanation ratio: the principal gradient explains 26%, 28%, 29%, and 29% of information for ages 1, 2, 4, and 6, respectively. **(c)** Histogram of the principal and secondary gradients at each age.

Structural connectome gradients were identified as eigenvectors of the similarity matrix of the SC from non-linear dimensionality reduction technique. The principal gradient exhibited a left-to-right axis, and the secondary gradient exhibited an anterior-to-posterior axis (Figure 3a, Supplementary Figure 1a). The principal gradient explained about 30% of information (Figure 3b, Supplementary Figure 1b). We also computed the structural gradients at 2, 4, and 6 years, and found they appear similar at ages 1, 2, 4, and 6 (Figure 3c).

Each gradient component (eigenvector) captures a different structural axis. Our principal gradient demonstrated a left-to-right hemispheric axis, primarily driven by weak interhemispheric connections, suggesting that it captures within-hemisphere connectivity patterns rather than interhemispheric integration. This spatial separation reflects the relative independence of left and right hemisphere connectivity architectures, with each hemisphere forming distinct connectivity due to limited inter-hemispheric structural connections. The secondary gradient exhibits an anterior-to-posterior axis, which typically reflects the transition from primary sensorimotor area located posteriorly to higher-order association and executive regions located anteriorly. This axis represents a structural network identity dimension that distinguishes regions based on their position in the brain's processing hierarchy, ranging from areas involved in basic sensory-motor processes to those supporting complex cognitive functions. These two structural gradient axes provide the anatomical foundation that constrains functional network organization. The hemisphere segregation axis may influence the efficiency of bilateral coordination required for many cognitive tasks (Herve et al., 2013), while the anterior-to-posterior axis reflects the structural scaffolding supporting the functional hierarchy from perception to cognition (Mahjoory et al., 2020).

To further examine within-hemisphere organization, we analyzed hemisphere-specific gradients by excluding inter-hemispheric connections and aligning the right hemisphere's structural gradients to their left hemisphere counterparts to facilitate hemisphere-wise comparison. In this analysis, the principal gradient (G1) showed an anterior-to-posterior axis, the secondary gradient (G2) a lateral-to-medial axis, and the third gradient (G3) a superior-to-inferior axis (Supplementary Figure 2). Notably, we observe an evident correspondence between these gradients and those reported in Park et al. (2021) with our G1 corresponding to E3, G2 to E1, and G3 to E2.

### 3.2 Individual prediction of IQ from structural gradients

**Figure 4:**
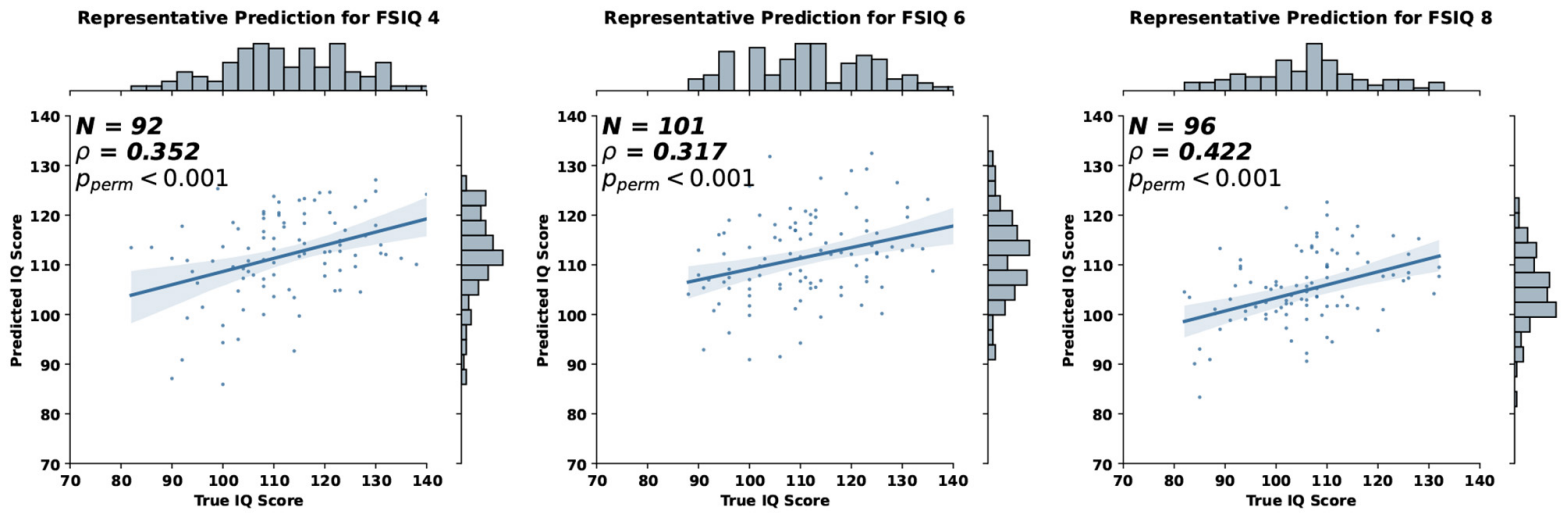
Representative prediction of full-scale IQ (FSIQ) at 4, 6, and 8 years from structural connectome gradients at age 1.

Using the proposed GCN-based approach, we were able to predict each individual's cognitive outcome from their structural gradients at age 1. Representative prediction results from our model are shown in Figure 4, where the statistical significance was assessed using permutation tests (Good, 2000; Manly, 2006), which provide exact p-values without distributional assumptions. The correlation between predicted and observed IQ scores was evaluated by shuffling predicted values 10,000 times to generate a null distribution under the hypothesis of no association.

We compared the prediction accuracy of our model with other existing machine learning methods in Tables 2, 3. We utilized the scikit-learn package in Python to implement other machine learning methods, using the default hyperparameters except for the following changes: for Random Forest, max_depth was set to 100 and n_estimators to 50; for MLP, hidden_layer_sizes was set to (64, 64, 64, 64). Compared to other methods, our model provided consistent and robust prediction results (mean Spearman's correlation > 0.25). We note that the results summarized in Tables 2, 3 are the average MAE, Spearman correlation, and Pearson correlation across 10 repetitions of 10-fold cross-validation. For each repetition, the results are aggregated across the 10 folds. In addition, we compared our model against a naïve baseline predictor that assigns the mean IQ of the training set to all test samples within each cross-validation fold. Our model exhibited competitive MAE values while showing substantial improvement in predictive correlation. These results demonstrate that the model captures meaningful individual differences in cognitive ability, with performance comparable to prior neuroimaging studies (*R* = 0.35–0.50 for general intelligence Vieira et al., 2022).

**Table 2:** Quantitative results for full-scale IQ (FSIQ) prediction at different ages where the input features are the principal and secondary gradients with Destrieux parcellation at age 1.

**Method**	**Mean absolute error (MAE)**			**Average Spearman correlation** ρ		
	**FSIQ4**	**FSIQ6**	**FSIQ8**	**FSIQ4**	**FSIQ6**	**FSIQ8**
naïve prediction	10.07 ± 0.03	10.23 ± 0.03	9.01 ± 0.04	−0.21 ± 0.05	−0.27 ± 0.03	−0.23 ± 0.07
SVR	9.97 ± 0.05	**10.17 ± 0.10**	**8.99 ± 0.11**	0.18 ± 0.02	0.18 ± 0.03	0.08 ± 0.07
Kernel Ridge	10.58 ± 0.31	11.75 ± 0.32	11.31 ± 0.39	0.19 ± 0.03	0.12 ± 0.04	−0.03 ± 0.06
MLP	10.40 ± 0.30	12.24 ± 0.39	11.23 ± 0.53	0.21 ± 0.03	0.05 ± 0.05	−0.07 ± 0.07
Random Forest	9.72 ± 0.19	10.35 ± 0.18	9.40 ± 0.19	0.22 ± 0.05	0.13 ± 0.05	−0.02 ± 0.07
GCN	**9.69 ± 0.57**	10.79 ± 0.26	9.53 ± 0.40	**0.36 ± 0.08**	**0.25 ± 0.05**	**0.33 ± 0.06**

The results with the best prediction accuracy were highlighted in bold. The second-best results were highlighted in underline. The standard deviations were computed over the 10 repetitions of cross-validations.

**Table 3:** Pearson correlation coefficients and their squared values (*r*^2^) for FSIQ prediction at different ages where the input features are the principal and secondary gradients with Destrieux parcellation at age 1.

**Method**	**Average Pearson correlation** ***r***			***r*** ^**2**^		
	**FSIQ4**	**FSIQ6**	**FSIQ8**	**FSIQ4**	**FSIQ6**	**FSIQ8**
naïve prediction	−0.21 ± 0.04^†^	−0.28 ± 0.03^†^	−0.22 ± 0.05^†^	0.04 ± 0.02	0.08 ± 0.02	0.05 ± 0.02
SVR	0.17 ± 0.02	0.19 ± 0.03	0.09 ± 0.06	0.03 ± 0.01	0.04 ± 0.01	0.01 ± 0.01
Kernel Ridge	0.27 ± 0.03	0.10 ± 0.04	−0.01 ± 0.06^†^	0.08 ± 0.02	0.01 ± 0.01	0.00 ± 0.00
MLP	0.23 ± 0.04	0.04 ± 0.04	−0.04 ± 0.06^†^	0.05 ± 0.02	0.01 ± 0.00	0.00 ± 0.00
Random Forest	0.22 ± 0.06	0.15 ± 0.04	−0.01 ± 0.09^†^	0.05 ± 0.03	0.02 ± 0.01	0.01 ± 0.01
GCN	**0.37 ± 0.07**	**0.25 ± 0.04**	**0.31 ± 0.05**	**0.15 ± 0.05**	**0.06 ± 0.02**	**0.10 ± 0.03**

The results with the best prediction accuracy were highlighted in bold. The second-best results were highlighted in underline. The standard deviations were computed over the 10 repetitions of cross-validations. Note that † indicates negative Pearson correlation.

We performed additional exploratory analyses with different numbers of gradient components as input features as well as employing the gradients computed from the AAL parcellation. The quantitative results are summarized in Supplementary Tables 2–4. Using only the principal gradient resulted in greater error and lower correlation at FSIQ4 and FSIQ8 compared to the models incorporating both primary and secondary gradients. For the AAL parcellation, inclusion of secondary gradient components improved correlation. Adding secondary gradients helped achieve a more stable training, resulting in consistently higher prediction accuracy across the IQ at different ages.

In addition to full-scale IQ, we examined prediction models for VIQ, NVIQ, and ABIQ. We trained the prediction model for each of the IQs, and the results are shown in Supplementary Table 5. Our model consistently predicted each individual's VIQ, NVIQ, and ABIQ at later ages with high prediction accuracy.

### 3.3 Regional relevance maps

Our machine learning methods were able to identify important features for predicting subsequent cognitive outcomes. Using Captum (Kokhlikyan et al., 2020), an explainable AI pytorch library that contains generic implementations of a variety of model gradient and perturbation-based attribution algorithms, we can determine the features relevant to our model's predictions.

**Figure 5:**
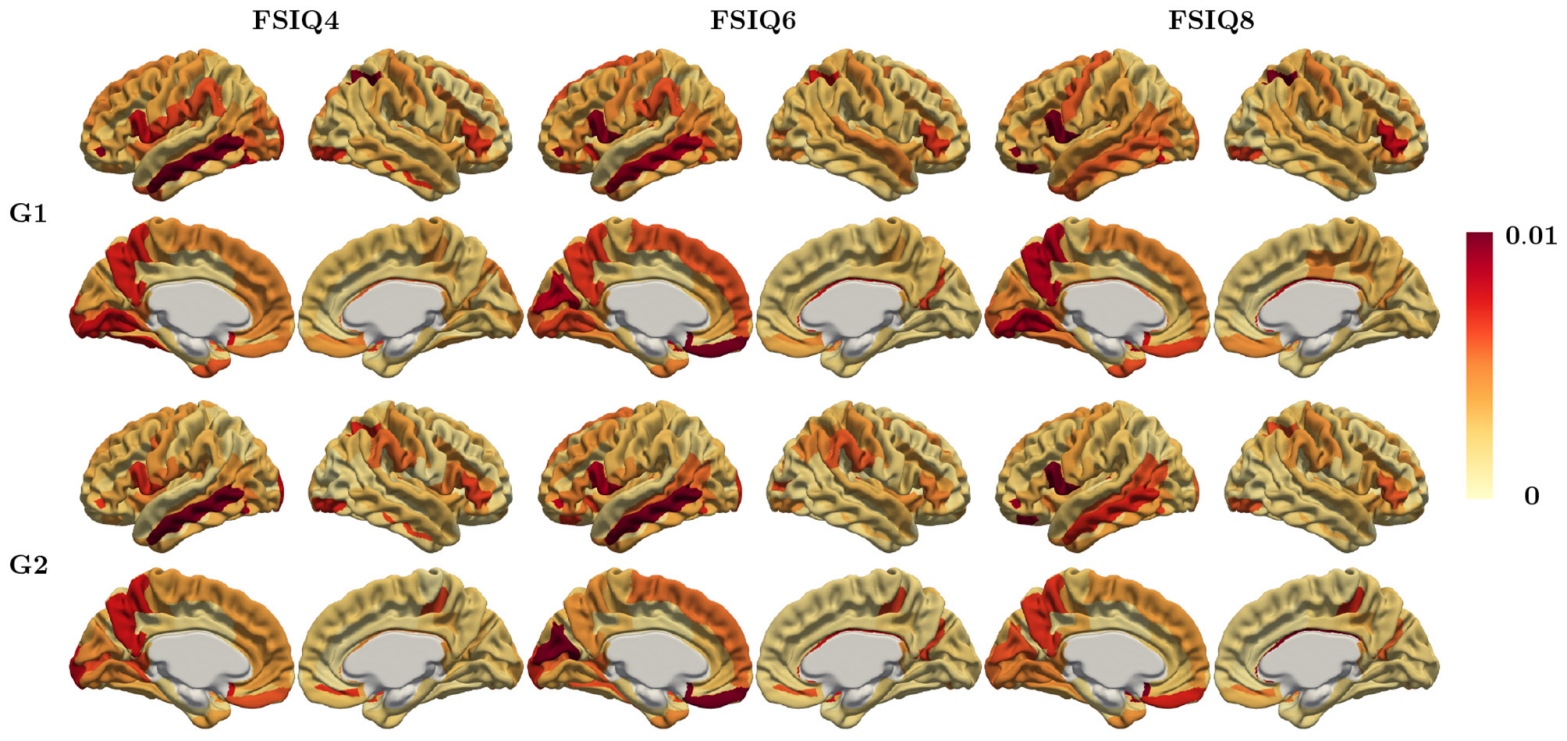
Regional relevance map averaged across all subjects using Saliency map method (Destrieux parcellation) for IQ at ages 4, 6, and 8.

The weight attributions of regional relevance for predicting each target IQ obtained by Saliency Maps (Simonyan, 2013) are shown in Figure 5, where the weights are averaged across different cross-validation folds for each region. It is important to note that individual feature importance maps may not always align perfectly with the average pattern. The regions consistently selected in the top 5% weights for all target IQ scores were found in the left hemisphere's lateral orbital sulcus, opercular part of the inferior frontal gyrus, and subcallosal gyrus, which are included in the FrontoParietal, CinguloOperc, and default mode network. We also applied other feature attribution methods, specifically Integrated Gradients (IG) (Sundararajan et al., 2017) as well as the feature attribution for AAL parcellation. The corresponding feature importance maps are shown in Supplementary Figures 3, 4, respectively.

It is crucial to note that the regional relevance maps in Figure 5, Supplementary Figures 3, 4 are widely distributed across the cortex. Regions with nearly zero weights were not highlighted, while only those with large magnitudes were emphasized. Although each feature attribution method and each parcellation provides a slightly different importance map, the regional relevance maps demonstrated remarkable consistency across FSIQ4, FSIQ6, and FSIQ8 measures including frontoparietal regions and other regions previously implicated in intelligence, with minor variations likely attributable to methodological differences rather than developmental changes. Notably, the relevance map derived from the principal gradient predominantly highlighted regions within the executive control network, encompassing areas crucial for working memory, attention control, and cognitive flexibility. In contrast, the secondary gradient's relevance map emphasized associative and sensory processing networks, reflecting regions essential for information integration and sensory-cognitive processing. These distinct patterns emphasize the complementary gradient roles.

## 4 Discussion

Using our approach, we found that WM connectome gradients at age 1 can be a stable, low-dimensional predictor of IQ at ages 4, 6, and 8. This suggests that the WM connectome, which matures in early childhood and appear similar at ages 1, 2, 4, and 6, is an important basis for individual variation of IQ in early and middle childhood and may be useful as an early imaging potential biomarker of future intelligence.

In our prediction model, regions identified by the feature attribution model were distributed across the frontal, temporal and parietal lobes, consistent with the distributed nature of intelligence detected in prior structural imaging studies (Jung and Haier, 2007; Basten et al., 2015; Deary et al., 2010). These regions include the left hemisphere's lateral orbital sulcus, opercular part of the inferior frontal gyrus, and subcallosal gyrus, and are part of the FrontoParietal, CinguloOperc, and default mode networks. Notably, several regions identified in the relevance maps correspond to hub nodes characterized by high degree centrality (Supplementary Figure 5). However, the relevance maps demonstrate greater selectivity compared to hub identification alone, indicating that the gradient-based approach captures functionally specific connectivity patterns embedded within the broader hub architecture. This selective engagement of hub regions based on their gradient-specific contributions provides a more neurobiologically meaningful framework for understanding intelligence than simply identifying highly connected nodes. We previously found that individually, high centrality WM connectome hubs were not related to IQ at 6 years (Bagonis et al., 2022), where the association was analyzed using partial correlation, with statistical significance adjusted through an FDR correction. In contrast, this study employed a machine learning prediction model that considers the whole brain network architecture. Another limitation of previous study is small sample sizes in age-specific comparisons. These small samples reduce statistical power to detect the true effects. The current analysis suggests that high centrality hubs are important for intelligence, but only in relation to the entire connectome.

While our study focused on general intelligence (IQ) as a comprehensive measure of cognitive outcome, exploring the structural connectome in relation to more specific cognitive subdomains is an important next step. For example, previous research from our group established that early verbal and nonverbal cognitive abilities were differentially predictive of later cognitive outcomes (Stephens et al., 2018). Further, these domain-specific differences in cognitive abilities may extend to regional connectivity patterns. For instance, left frontal connectivity may predict verbal more strongly compared to non-verbal intelligence (Suprano et al., 2020). Future studies employing comprehensive neuropsychological batteries that assess specific cognitive subdomains would provide a more nuanced understanding of how different properties of the structural connectome relate to diverse aspects of cognitive development.

In addition to the conventional sensorimotor-to-transmodal axis derived from functional connectome gradients, anatomically-derived structural gradients provide different information about individual variations of cortical organization, and may help identify variance in cognitive outcomes relevant to the functions organized along those gradients. Regions at one end of an anterior-posterior gradient such as frontal areas which are connected with subcortical regions such as the striatum and thalamus, potentially influencing foundational processes (Tekin and Cummings, 2002; Rae et al., 2015), while regions at the other end such as the parietal and occipital areas tend to be related to particular functions such as visuospatial processing, sensorimotor integration, or memory (Rolls et al., 2023; Dalton et al., 2022).

Our graph convolutional neural network (GCN)-based prediction model demonstrated consistent and robust predictive performance from age 1 (mean absolute error 9.5–10.8, mean Spearman correlation 0.3–0.4). Additional experiments confirm that our method is robust with the different parcellation and different number of gradient components as input features. Using the principal gradient as the sole input feature yielded comparable prediction results, though it was less stable compared to using secondary gradients as an additional input feature.

We noted the challenges associated with explaining positive and negative relevance maps from IG methods in the context of a regression task. Interpreting the signed values of regional relevance maps can be quite ambiguous, and the choice to use the absolute values of the relevance map is influenced by the specific characteristics of the dataset (Smilkov et al., 2017). Additionally, we observed that some regions in Supplementary Figures 3, 4 displayed different signs. It is also important to mention that the input SC gradients are unitless and indicate relative positions along the computed axes. Therefore, we present the raw relevance maps but focus on their magnitudes when identifying the most important regions.

Previous studies have identified different cortical connectivity gradients from different datasets and in cohorts of different ages (Margulies et al., 2016; Paquola et al., 2019; Vos de Wael et al., 2021; Xia et al., 2022; He et al., 2024). This suggests that observed gradients are very sensitive to differences in acquisition and analytic methodologies, as well as subject variables. Indeed, it has recently been argued that the conceptualization of smooth, continuous gradients is not supported by what is known about the neurobiological basis of discrete cortical realization in the human cortex (Petersen et al., 2024). Cortical gradients may be telling us something useful about cortical organization and its relationship to behavior, but future research is needed to figure out exactly what it is.

Structural connectivity was derived by quantifying the relative number of streamlines, which serves as a measure of connectivity strength. Structural connectivity quantification via streamline counting has known limitations, such as reduced streamline counts due to motion during acquisition, or due to passing through brain regions with multiple crossing fibers. Additionally, the choice of cortical parcellations (Destrieux or AAL) influences both the spatial resolution and regional boundaries used to define network nodes, which may affect gradient topography and the specific regions identified as most predictive, though our results demonstrated robustness across these different parcellation approaches. Further, the use of different kernels or gradient templates may yield different or flipped gradient axes (Supplementary Figure 6). Bajada et al. highlighted the importance of the similarity metric (Bajada et al., 2020). Cosine similarity, a commonly used measure, ranges from -1 to 1 and is less sensitive to small angular differences between vectors. In contrast, normalized angle similarity, derived by applying the inverse cosine function to cosine similarity, ranges from 0 to 1 and provides a direct measure of angular differences. In the future, we plan to explore alternative methods for reconstructing structural connectivity and analyzing connectome gradients.

In our machine learning prediction model, we did not consider any demographic information such as maternal education, gestational age at birth, or gender. It is widely known that maternal education is highly associated with the child's cognitive outcome, as mothers with higher education typically provide richer language environments, more learning resources at home, and stronger educational values that directly support brain development and cognitive growth (Bradley and Corwyn, 2002; Noble et al., 2015). We applied a linear regression model to demographic information and found that it outperformed imaging features for IQ prediction in our sample (Supplementary Table 7). This finding is consistent with previous studies showing that demographic variables often equal or exceed neuroimaging-based predictions, particularly with modest sample sizes (Vieira et al., 2022). In the future, we will perform additional analyses to investigate whether the WM connectome mediates the association between maternal education and cognitive ability (Baron and Kenny, 1986). We will also utilize the demographics as additional features in the prediction model to examine whether they have a higher predictive power than the WM connectome only.

A limitation of our prediction model relates to the harmonization procedure. We applied ComBat harmonization to the structural gradients using the combined training and testing data for each cross-validation fold. Ideally, ComBat should be applied separately to training and testing sets to avoid potential data leakage; however, our limited sample sizes made separate harmonization of individual testing sets statistically unreliable. To address this concern, we conducted sensitivity analyses by training prediction models without ComBat harmonization, which yielded comparable results to the harmonized models (Supplementary Table 6), suggesting that this methodological choice did not substantially influence our findings.

Recently, multi-scale structural connectome gradients were constructed using microstructural similarity and cortico-cortical proximity as well as white matter tractography (He et al., 2024). The identified multi-scale/multi-modal gradients were consistent with the well-known primary-to-association and anterior-posterior gradients. These multi-scale connectome gradients may open up new possibilities for uncovering the comprehensive principles of cortical network organization during brain development. In the future, we plan to generate such multi-scale gradients in our dataset and investigate the developmental changes and the association to functional specialization in early childhood.

In summary, in this work, we identified structural gradients at age 1 and showed their association with cognitive outcomes. We developed a machine learning model to predict IQ scores by utilizing the structural gradients as input features. Our model demonstrated moderate prediction accuracy in predicting IQs at later ages. These findings suggest that the structural gradients serve as compact and interpretable representations of complex brain networks, effectively capturing individual differences in early childhood.

## Data Availability

Publicly available datasets were analyzed in this study. This data can be found here: The EBDS dataset is publicly available through the NIMH Data Archive (https://nda.nih.gov) and the code is publicly available at: https://github.com/yoonmihong/SCgradients.
