# Supplementary material for: Structural connectome gradients and their relationship to IQ in childhood
Source: Front Hum Neurosci. 2025 Nov 24;19:1688296. doi: 10.3389/fnhum.2025.1688296 (PMC12682798; doi:10.3389/fnhum.2025.1688296)
Supplement: Supplementary file 1 [file Data_Sheet_1.pdf]

Supplementary Material

Yoonmi Hong[1,*], Emil Cornea[1], Jessica B. Girault[1,2], Rebecca L. Stephens[1], Maria Bagonis[3], Mark Foster[1], Sun Hyung Kim[1], Juan Carlos Prieto[1], Martin A. Styner[1,4,5], and John H. Gilmore[1]

1 Department of Psychiatry, University of North Carolina at Chapel Hill, NC, U.S.A.

2 Carolina Institute for Developmental Disabilities, University of North Carolina at Chapel Hill, NC, U.S.A.

3 National Institute of Disorders and Stroke (NINDS) at the NIH

4 Department of Computer Science, University of North Carolina at Chapel Hill, NC, U.S.A.

5 Department of Biomedical Engineering, University of Basel, Switzerland

***** Corresponding author:

Name Yoonmi Hong
Department Department of Psychiatry
Institute University of North Carolina at Chapel Hill
E-mail yoonmi_hong@med.unc.edu

Table S1: Participant demographic and scan information

	Overall Cohort (N=388)			
	Mean (SD)			
Gestational age at birth (days)	261.15 (18.72)			
Birth weight (g)	2845.54 (713.44)			
Maternal education (years)	14.9 (3.3)			
	N (%)			
Sex, male	201 (52%)			
Twin	200 (52%)			
Non High-risk Singleton	141 (36%)			
High-risk Singleton[a]	47 (11%)			
	1 year N=173	2 year N=152	4 year N=136	6 year N=160
	Mean (SD)	Mean (SD)	Mean (SD)	Mean (SD)
Age at scan (months)	12.99 (0.95)	25.11 (1.03)	48.94 (1.33)	73.02 (1.47)
Number of excluded DWIs	1.46 (1.92)	1.11 (1.90)	4.89 (3.57)	3.32 (3.12)
Number of DWIs with large translation (> 1mm)	0.40 (1.16)	0.89 (3.71)	1.09 (1.58)	0.87 (1.51)
	N(%)	N(%)	N(%)	N(%)
Allegra	125 (72%)	92 (61%)	17 (13%)	28 (17%)
Tim Trio	48 (28%)	60 (39%)	119 (87%)	132 (83%)

[a]Maternal diagnosis: Schizophrenia (14), Schizoaffective disorder (4), Psychosis NOS (1), Bipolar (9), Mood disorder NOS (11), other/unable to determine (8); NOS: not otherwise specified

Table S2: Quantitative results for IQ prediction at different ages where the input feature is the principal gradient with Destrieux parcellation at age 1. The results with the best prediction accuracy were highlighted in bold. The second best results were highlighted in underline.

	Mean Absolute Error (MAE)			Average Spearman Correlation		
	FSIQ4	FSIQ6	FSIQ8	FSIQ4	FSIQ6	FSIQ8
SVR	$\underline{10.12 \pm 0.11}$	$\underline{10.44 \pm 0.09}$	$\underline{9.35 \pm 0.14}$	0.07 ± 0.04	0.07 ± 0.03	-0.01 ± 0.06
Kernel Ridge	10.17 ± 0.32	11.46 ± 0.24	10.95 ± 0.42	$\underline{0.22 \pm 0.05}$	$\underline{0.13 \pm 0.04}$	$\underline{0.02 \pm 0.07}$
MLP	10.97 ± 0.26	12.30 ± 0.41	11.32 ± 0.32	0.17 ± 0.03	0.01 ± 0.05	-0.05 ± 0.05
Random Forest	$\mathbf{9.96 \pm 0.20}$	10.53 ± 0.19	$\mathbf{9.25 \pm 0.10}$	0.20 ± 0.07	0.05 ± 0.05	0.01 ± 0.05
GCN	10.49 ± 0.29	$\mathbf{10.16 \pm 0.34}$	9.86 ± 0.47	$\mathbf{0.23 \pm 0.04}$	$\mathbf{0.31 \pm 0.05}$	$\mathbf{0.26 \pm 0.06}$

Table S3: Quantitative results for IQ prediction at different ages where the input features are the principal and secondary gradients with AAL parcellation at age 1. The results with the best prediction accuracy were highlighted in bold. The second best results were highlighted in underline.

	MAE			Average Spearman Correlation		
	FSIQ4	FSIQ6	FSIQ8	FSIQ4	FSIQ6	FSIQ8
SVR	$\underline{10.12 \pm 0.07}$	$\mathbf{9.88 \pm 0.09}$	$\mathbf{8.60 \pm 0.11}$	0.09 ± 0.03	$\mathbf{0.20 \pm 0.03}$	$\mathbf{0.24 \pm 0.05}$
Kernel Ridge	10.28 ± 0.17	11.28 ± 0.33	9.99 ± 0.25	$\underline{0.20 \pm 0.02}$	$\underline{0.11 \pm 0.04}$	0.17 ± 0.05
MLP	10.63 ± 0.33	12.43 ± 0.61	11.00 ± 0.52	0.16 ± 0.05	0.07 ± 0.05	0.12 ± 0.06
Random Forest	$\mathbf{10.05 \pm 0.18}$	$\underline{10.30 \pm 0.17}$	$\underline{9.18 \pm 0.28}$	0.14 ± 0.04	0.09 ± 0.05	0.09 ± 0.08
GCN	10.62 ± 0.34	12.31 ± 0.26	10.48 ± 0.37	$\mathbf{0.25 \pm 0.03}$	0.07 ± 0.03	$\underline{0.22 \pm 0.04}$

Table S4: Quantitative results for IQ prediction at different ages where the input feature is the principal gradient with AAL parcellation at age 1. The results with the best prediction accuracy were highlighted in bold. The second best results were highlighted in underline.

	MAE			Average Spearman Correlation		
	FSIQ4	FSIQ6	FSIQ8	FSIQ4	FSIQ6	FSIQ8
SVR	$\mathbf{10.16 \pm 0.10}$	$\mathbf{10.63 \pm 0.19}$	$\underline{9.76 \pm 0.16}$	0.03 ± 0.03	-0.12 ± 0.06	-0.03 ± 0.07
Kernel Ridge	10.31 ± 0.13	11.58 ± 0.31	10.58 ± 0.28	$\underline{0.18 \pm 0.02}$	$\mathbf{0.01 \pm 0.03}$	$\underline{0.11 \pm 0.06}$
MLP	10.89 ± 0.44	12.28 ± 0.55	12.01 ± 0.44	0.14 ± 0.05	-0.05 ± 0.05	-0.05 ± 0.04
Random Forest	$\underline{10.25 \pm 0.29}$	$\underline{10.87 \pm 0.18}$	$\mathbf{9.53 \pm 0.21}$	0.11 ± 0.09	-0.11 ± 0.07	-0.03 ± 0.08
GCN	10.41 ± 0.45	11.88 ± 0.46	10.21 ± 0.38	$\mathbf{0.22 \pm 0.06}$	$\mathbf{0.01 \pm 0.07}$	$\mathbf{0.15 \pm 0.04}$

Table S5: Quantitative results for IQ prediction of different subscales assessed at ages 4, 6, and 8 where the input features are the principal and the secondary gradients with Destrieux parcellation at age 1 and the prediction model is GCN.

	MAE			Average Spearman Correlation		
	4 year	6 year	8 year	4 year	6 year	8 year
VIQ	13.33 ± 0.59	13.03 ± 0.46	10.24 ± 0.68	0.28 ± 0.05	0.16 ± 0.05	0.31 ± 0.08
NVIQ	9.82 ± 0.43	11.34 ± 0.43	12.04 ± 0.53	0.16 ± 0.07	0.26 ± 0.06	0.17 ± 0.07
ABIQ	10.02 ± 0.69	12.11 ± 0.77	11.55 ± 0.46	0.24 ± 0.07	0.14 ± 0.09	0.18 ± 0.05

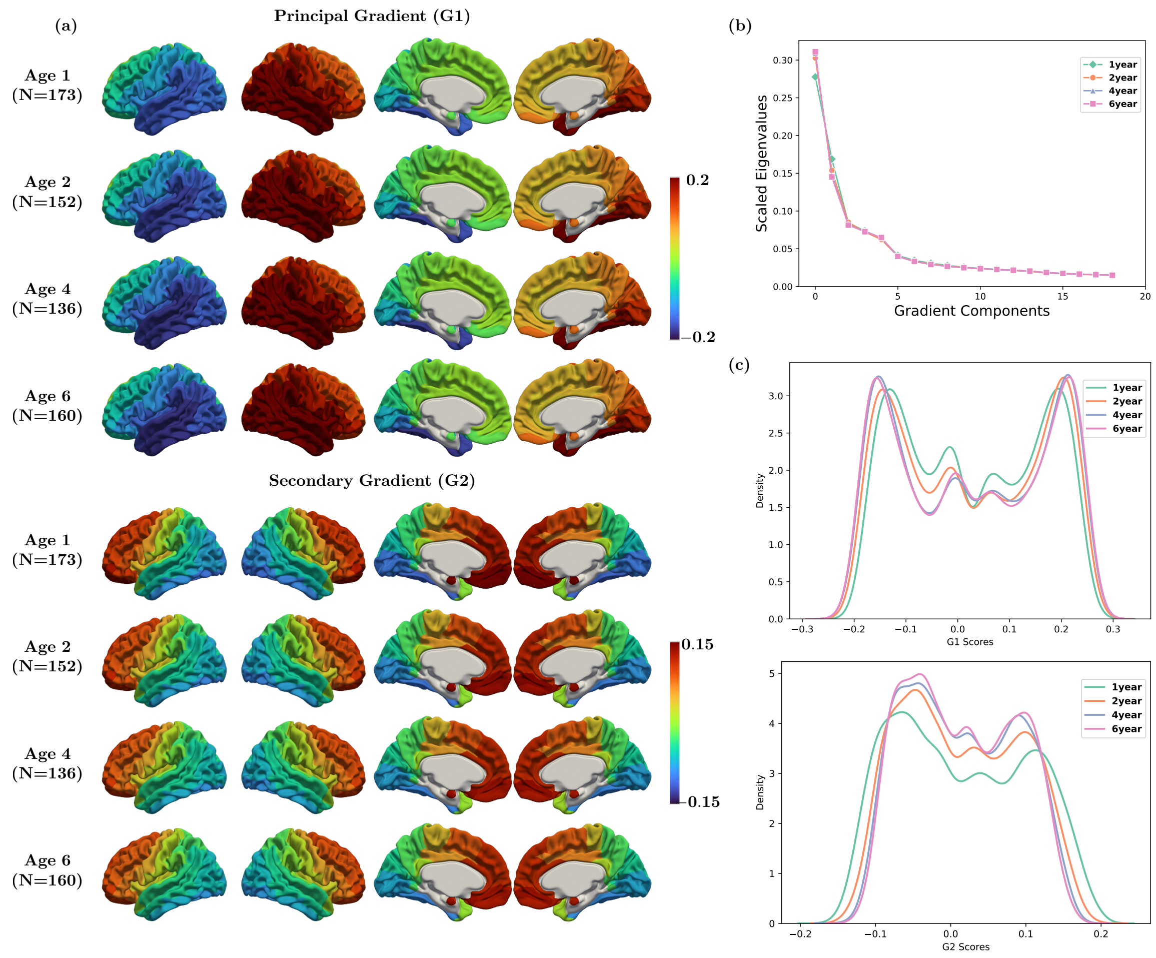

Figure S1: (a) Average structural gradients at each age using AAL parcellation (b) Explanation ratio: the principal gradient explains 28%, 30%, 31%, and 31% of information for ages 1, 2, 4, and 6, respectively. (c) Histogram of the principal and secondary gradients at each age.

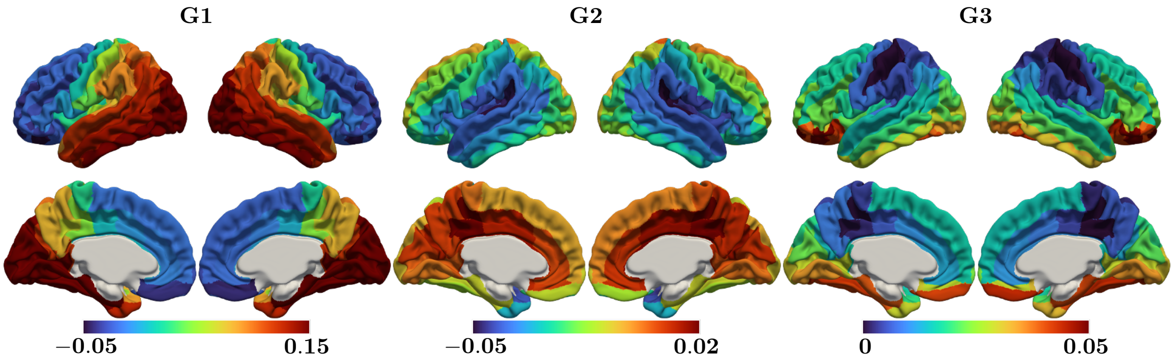

Figure S2: Hemisphere-specific gradients at age 1 (Destrieux parcellation)

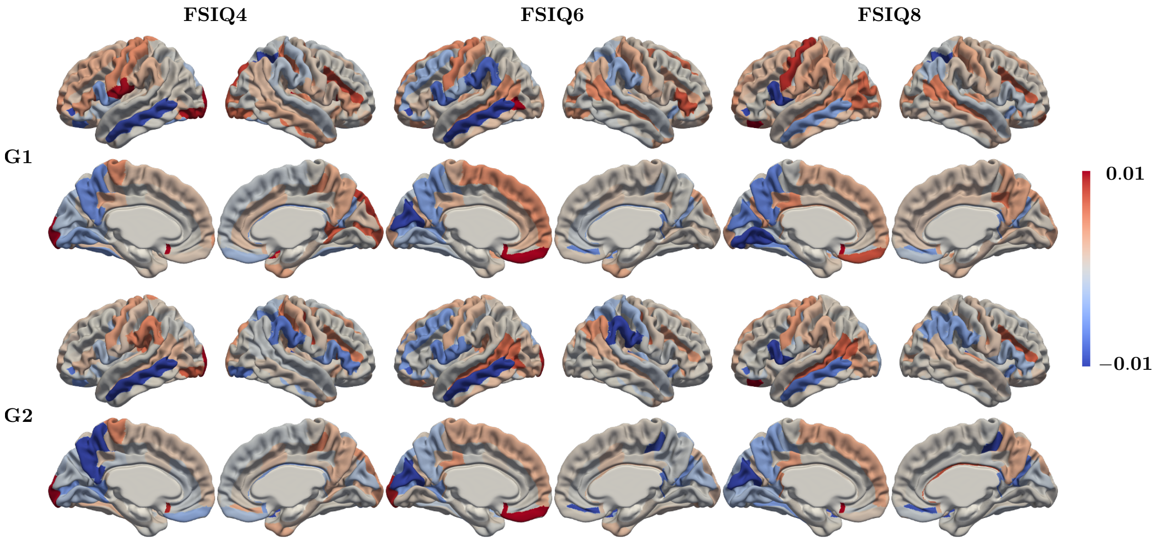

Figure S3: Regional relevance map averaged across all subjects using IG method (Destrieux parcellation) for IQ at ages 4, 6, and 8.

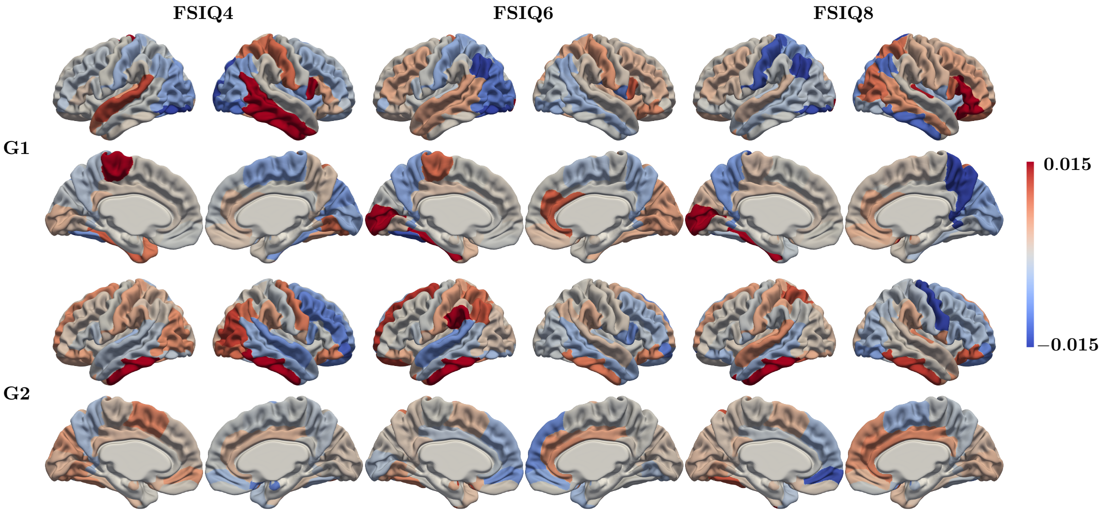

Figure S4: Regional relevance map averaged across all subjects using IG method (AAL parcellation) for IQ at ages 4, 6, and 8.

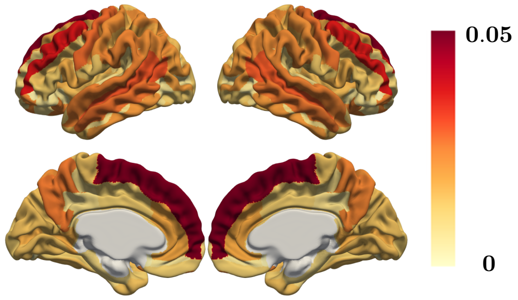

Figure S5: Degree centrality at age 1 (Destrieux parcellation)

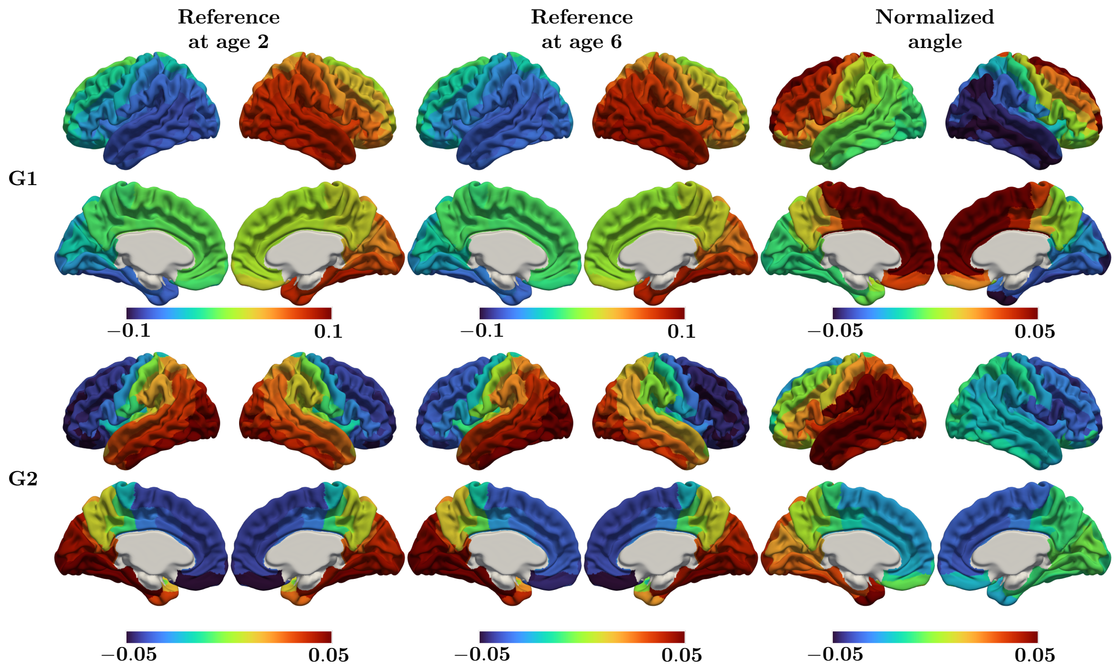

Figure S6: Principal and secondary gradients constructed from different reference subjects as a gradient template and normalized angle kernel (Destrieux parcellation)

Table S6: Quantitative results for IQ prediction at different ages where the input features are the principal and secondary gradients with Destrieux parcellation at age 1 without applying ComBat and the prediction model is GCN.

MAE			Average Spearman Correlation		
4 year	6 year	8 year	4 year	6 year	8 year
10.13 ± 0.33	10.69 ± 0.29	9.34 ± 0.28	0.29 ± 0.04	0.29 ± 0.04	0.31 ± 0.04

Table S7: Quantitative results for IQ prediction at ages 4, 6, and 8 where the input features are demographics information and the prediction model is a linear regression.

	MAE			Average Spearman Correlation		
	4 year	6 year	8 year	4 year	6 year	8 year
Maternal education	9.04 ± 0.07	8.75 ± 0.04	8.10 ± 0.07	0.46 ± 0.01	0.48 ± 0.01	0.49 ± 0.01
Maternal education, Sex, Gestational Age at Birth	9.14 ± 0.10	8.94 ± 0.08	8.25 ± 0.12	0.45 ± 0.01	0.49 ± 0.01	0.50 ± 0.01
